# Sugar-Sweetened Beverage Consumption Among Adults — 18 States, 2012

**Published:** 2014-08-15

**Authors:** Gayathri S. Kumar, Liping Pan, Sohyun Park, Seung Hee Lee-Kwan, Stephen Onufrak, Heidi M. Blanck

**Affiliations:** 1 Epidemic Intelligence Service, Office of Surveillance, Epidemiology, and Laboratory Services, CDC; 2 Division of Nutrition, Physical Activity, and Obesity, National Center for Chronic Disease Prevention and Health Promotion, CDC

Reducing consumption of calories from added sugars is a recommendation of the 2010 Dietary Guidelines for Americans* and an objective of *Healthy People 2020*.† Sugar-sweetened beverages (SSB) are major sources of added sugars in the diets of U.S. residents (1). Daily SSB consumption is associated with obesity and other chronic health conditions, including diabetes and cardiovascular disease (2). U.S. adults consumed an estimated average of 151 kcal/day of SSB during 2009–2010, with regular (i.e., nondiet) soda and fruit drinks representing the leading sources of SSB energy intake (3,4). However, there is limited information on state-specific prevalence of SSB consumption. To assess regular soda and fruit drink consumption among adults in 18 states, CDC analyzed data from the 2012 Behavioral Risk Factor Surveillance System (BRFSS). Among the 18 states surveyed, 26.3% of adults consumed regular soda or fruit drinks or both ≥1 times daily. By state, the prevalence ranged from 20.4% to 41.4%. Overall, consumption of regular soda or fruit drinks was most common among persons aged 18–34 years (24.5% for regular soda and 16.6% for fruit drinks), men (21.0% and 12.3%), non-Hispanic blacks (20.9% and 21.9%), and Hispanics (22.6% and 18.5%). Persons who want to reduce added sugars in their diets can decrease their consumption of foods high in added sugars such as candy, certain dairy and grain desserts, sweetened cereals, regular soda, fruit drinks, sweetened tea and coffee drinks, and other SSBs. States and health departments can collaborate with worksites and other community venues to increase access to water and other healthful beverages.§

BRFSS is an annual, state-based, random-digit–dialed landline and cell phone survey of U.S. adults (aged ≥18 years) that assesses the prevalence of preventive health practices and risk factors for chronic diseases and other conditions.¶ It uses a complex, multistage cluster sampling design to select a sample representing the civilian noninstitutionalized U.S. adult population in the 50 states, District of Columbia, and three U.S. territories. Weighting is used to adjust for nonresponse, noncoverage, and differences in probably of selection. The median response rate for the 18 states included in this report was 46.2% (range = 27.7%–60.4%).**

In 2012, BRFSS included an optional module with questions about SSB consumption: “During the past 30 days, how often did you drink regular soda or pop that contains sugar? Do not include diet soda or diet pop.” and ”During the past 30 days, how often did you drink sweetened fruit drinks, such as Kool-Aid, cranberry juice cocktail, and lemonade? Include fruit drinks you made at home and added sugar to.” Respondents could report monthly, weekly, or daily consumption. All responses were subsequently converted to daily consumption. Daily intake of regular soda, fruit drinks, or both was calculated by summing the daily frequencies for regular soda and fruit drinks. Responses were categorized as none, <1 time/day, and ≥1 times/day. A total of 115,291 adults from the 18 states that offered the module responded to the SSB questions. A total of 1,900 respondents with missing responses to either the regular soda or fruit drink questions were excluded, leaving an analytic sample of 113,391 adults. Chi-square tests were used to determine whether regular soda and fruit drink consumption differed by age group, sex, and race/ethnicity for each state, with p<0.05 as the criterion for statistical significance. Estimates were not reported if a sample size was <50 or the relative standard error was ≥30%.

In 2012, 26.3% of respondents reported consuming regular soda, fruit drinks, or both ≥1 times daily (17.1% for regular soda and 11.6% for fruit drinks). The prevalence among states was highest in Mississippi (41.4%), followed by Tennessee (39.2% ) (Table 1). The prevalence of regular soda consumption ≥1 times daily was highest in Mississippi (32.4%) and Tennessee (30.2%), and the prevalence of fruit drink consumption was highest in Nevada (18.7%), Mississippi (17.0%), and Tennessee (16.5%).

Overall, regular soda and fruit drink consumption ≥1 times daily was most common among persons aged 18–34 years (24.5% and 16.6% for daily regular soda and fruit drink consumption, respectively), men (21.0% and 12.3%), non-Hispanic blacks (20.9% and 21.9%), and Hispanics (22.6% and 18.5%) (Table 2). In most states, regular soda consumption was most common among persons aged 18–34 years and men. Mississippi and Tennessee had the highest prevalence of regular soda consumption ≥1 times daily among those aged 18–34 years (47.4% and 40.0%, respectively) and men (36.8% and 33.7%).

In most states, fruit drink consumption ≥1 times daily was most common among persons aged 18–34 years, non-Hispanic blacks, and Hispanics (Table 3). Mississippi and Nevada had the highest prevalence among those aged 18–34 years (28.7% and 26.6%, respectively). Tennessee and Nevada had the highest prevalence among non-Hispanic blacks (30.5% and 28.7%, respectively). Nevada and Nebraska had the highest prevalence among Hispanics (33.8% and 27.8%%, respectively).

## Discussion

In 2012, about one in four adults reported consuming regular soda, fruit drinks, or both ≥1 times daily in the 18 states surveyed. The states with the highest prevalence of daily consumption of regular soda, fruit drinks, or both were Mississippi and Tennessee. Further, daily regular soda and fruit drink consumption was most common among those aged 18–34 years, men, non-Hispanic blacks, and Hispanics. Reducing SSB consumption as part of a healthy lifestyle might help with weight management and reduce the risk for chronic diseases among U.S. adults. Persons who want to reduce their daily added sugar intake can consider replacing their consumption of SSB with healthier drinking options (e.g., water, unsweetened tea, and fat-free milk).

These data from respondents in the 18 states that administered the optional SSB module as part of BRFSS in 2012 indicated that 26.3% of U.S. adults drank regular soda, fruit drinks or both daily. In contrast, data from the National Health and Nutrition Examination Survey (NHANES) indicated that the prevalence of daily SSB consumption (including all types of SSB) during 2007–2008 ranged from 50% for adults aged ≥35 years to 73% for adults aged 20–34 years (3). Possible reasons for this discrepancy include the following: 1) NHANES used 24-hour dietary recall whereas BRFSS used 30-day recall; 2) other SSB types such as sports and energy drinks, which contribute about 4%–8% of total SSB intake on a given day (3), were counted by NHANES but not by BRFSS; 3) NHANES is an in-person survey whereas BRFSS is conducted by telephone; 4) NHANES response rates are generally higher than BRFSS response rates††; and 5) the NHANES data were collected 4–5 years before the BRFSS data; regular soda and fruit drink consumption among adults aged ≥20 years has been decreasing nationally over the last decade (3,4).

The reasons for higher SSB consumption in certain states (e.g., Mississippi and Tennessee) are unclear. It could result from differences in the food environment and beverage marketing. For example, supermarkets in the southern region of the United States apportion more advertising space in sales circulars to SSB than do supermarkets in other regions, possibly increasing likelihood of SSB consumption (5). A previous study reported that the diet quality of adults in the lower Mississippi Delta, assessed by the Healthy Eating Index, was lower than other areas of the United States (6). This difference could be attributed to lower socioeconomic status, cultural factors, and food availability and accessibility in the area.

Somewhat similar to the present study, other researchers also have reported that younger adults (aged 20–34 years), men, non-Hispanic blacks, and Hispanics are more likely to consume SSB daily (3,4) compared with others. Possible reasons why these groups consume SSB more often might include taste preference, family influence, eating outside of the home, greater exposure to SSB marketing,§§ availability and affordability of SSB in particular communities or neighborhoods, and limited knowledge of the caloric content of SSB and their potential contribution to obesity (7,8). For example, the proportion of adults who knew the approximate calorie content of a 24-ounce soda was lowest among non-Hispanic blacks (8). Another explanation for higher SSB consumption could be lower health literacy in some subpopulations, especially among men and blacks (9). Further research could help identify why these disparities exist and how they might be addressed.

The findings in this report are subject to at least five limitations. First, estimates of regular soda and fruit drink consumption were based on self-report, and respondents might not have accurately reported their consumption; therefore, estimates might be either underestimated or overestimated. Second, the consumption frequency of only two types of SSB (regular soda and fruit drinks) was assessed; other types of SSB (e.g., sports and energy drinks, sweetened tea, and coffee drinks) were not included. Third, though it was possible to estimate the prevalence of the frequency of intake as SSB consumption per day, it was not possible to determine the actual amount of SSB consumed. Therefore, the daily calories from SSB could not be determined. Fourth, response bias might have affected the results because survey response rates ranged from 27.6% to 60.4% among states. Finally, these analyses were limited to adults in the 18 states with SSB data available, which limits the generalizability of the findings to the entire U.S. adult population.

SSB such as regular soda and fruit drinks contain added sugars and are sources of calories but have few, if any, essential nutrients (3,4). Because of the potential adverse impact of SSB consumption on diet quality, obesity and other chronic health conditions (2), reducing SSB consumption as part of a healthy lifestyle might help with weight management and the reduction of chronic diseases among U.S. adults. These findings among 18 states suggest that certain segments of the U.S. adult population consume regular soda and fruit drinks more often than others. Persons who want to reduce added sugars in their diet can decrease their consumption of regular soda and fruit drinks, which are the leading sources of SSB (3,4). States and health departments can support persons in these efforts by developing educational campaigns to inform consumers about beverage options and by helping worksites and other community venues increase access to healthful beverages such as water (10).

What is already known on this topic?

Sugar-sweetened beverages (SSB) are major sources of added sugars and calories in U.S. diets, and daily SSB consumption has been associated with obesity, diabetes, and cardiovascular disease. During 2009–2010, U.S. adults consumed an average of 151 kcal/day of SSB, with regular soda and fruit drinks representing the leading sources of SSB energy intake.

What is added by this report?

This is the first state-specific report on daily SSB intake. Among the 18 participating states, the states with the highest prevalence of consumption of regular soda, fruit drinks, or both were Mississippi and Tennessee. Overall, daily regular soda and fruit drink consumption was most common among those aged 18–34 years, men, non-Hispanic blacks, and Hispanics.

What are the implications for public health practice?

The findings from this study suggest that certain segments of the U.S. adult population consume regular soda and fruit drinks more often than others, which might contribute to weight gain and other chronic conditions. States and health departments can support persons deciding to reduce their regular soda and fruit drink consumption through activities that educate and inform consumers about beverage options and that help worksites and other community venues increase access to healthful beverages.

## Figures and Tables

**TABLE 1:** Prevalence* of regular soda† or fruit drink consumption among adults, by state — Behavioral Risk Factor Surveillance System, 18 states, 2012

	Consumption of regular soda, fruit drinks, or both			Regular soda consumption			Fruit drink consumption		
			
	None	<1 time/day	≥1 times/day	None	<1 time/day	≥1 times/day	None	<1 time/day	≥1 times/day
									
State (no. respondents)	% (95% CI)	% (95% CI)	% (95% CI)	% (95% CI)	% (95% CI)	% (95% CI)	% (95% CI)	% (95% CI)	% (95% CI)
**Overall** (**11,3391)**	**28.5** (**27.8–29.2)**	**45.2** (**44.4–46.1)**	**26.3** (**25.5–27.0)**	**41.6** (**40.8–42.4)**	**41.3** (**40.5–42.2)**	**17.1** (**16.5–17.7)**	**52.8** (**52.0–53.6)**	**35.6** (**34.8–36.4)**	**11.6** (**11.0–12.2)**
California (3,998)	29.0 (27.0–30.9)	48.1 (45.9–50.4)	22.9 (20.9–24.9)	42.8 (40.6–45.0)	44.0 (41.7–46.3)	13.2 (11.6–14.8)	48.2 (46.0–50.4)	41.8 (39.5–44.0)	10.0 (8.6–11.5)
Delaware (5,025)	29.6 (28.0–31.2)	43.8 (41.9–45.7)	26.6 (24.8–28.4)	41.6 (39.7–43.4)	40.7 (38.8–42.6)	17.7 (16.1–19.3)	55.3 (53.4–57.3)	33.1 (31.3–35.0)	11.5 (10.2–12.9)
Georgia (5,410)	24.1 (22.6–25.6)	42.8 (40.9–44.7)	33.1 (31.3–35.0)	36.4 (34.7–38.2)	40.7 (38.8–42.5)	22.9 (21.2–24.6)	51.6 (49.7–53.5)	34.2 (32.3–36.0)	14.2 (12.8–15.6)
Hawaii (7,152)	32.0 (30.4–33.6)	47.6 (45.8–49.4)	20.4 (18.9–21.9)	44.7 (42.9–46.4)	43.2 (41.5–45.0)	12.1 (10.9–13.3)	57.0 (55.2–58.5)	32.9 (31.2–34.6)	10.1 (8.9–11.2)
Iowa (3,277)	28.3 (26.5–30.1)	43.4 (41.3–45.6)	28.3 (26.2–30.4)	39.6 (37.6–41.7)	38.1 (36.0–40.2)	22.2 (20.3–24.2)	59.1 (57.0–61.3)	33.1 (31.0–35.2)	7.8 (6.5–9.1)
Kansas (5,616)	27.2 (25.8–28.6)	42.5 (40.8–44.3)	30.3 (28.5–32.0)	36.5 (34.9–38.2)	39.7 (37.9–41.4)	23.8 (22.1–25.5)	59.2 (57.4–61.1)	31.2 (29.4–32.9)	9.6 (8.4–10.8)
Maryland (5,760)	29.7 (27.8–31.6)	46.9 (44.5–49.3)	23.4 (21.2–25.6)	42.6 (40.3–44.8)	44.0 (41.6–46.4)	13.4 (11.7–15.2)	50.8 (48.5–53.2)	38.9 (36.5–41.3)	10.3 (8.7–11.9)
Minnesota (11,224)	27.8 (26.8–28.9)	47.8 (46.6–49.1)	24.4 (23.2–25.5)	39.8 (38.6–41.0)	42.3 (41.0–43.5)	17.9 (16.9–19.0)	56.5 (55.3–57.8)	35.5 (34.3–36.7)	8.0 (7.2–8.7)
Mississippi (7,242)	23.3 (22.0–24.6)	35.2 (33.7–36.8)	41.4 (39.8–43.1)	30.5 (29.0–31.9)	37.1 (35.5–38.7)	32.4 (30.7–34.1)	56.9 (55.2–58.6)	26.1 (24.6–27.6)	17.0 (15.6–18.4)
Montana (8,154)	29.8 (28.5–31.0)	47.5 (46.1–49.0)	22.7 (21.4–24.0)	41.2 (39.8–42.6)	43.0 (41.5–44.4)	15.8 (14.7–16.9)	60.4 (59.0–61.8)	30.9 (29.6–32.3)	8.7 (7.8–9.5)
Nebraska (11,709)	25.4 (24.3–26.4)	45.8 (44.5–47.0)	28.9 (27.7–30.0)	37.4 (36.2–38.5)	40.9 (39.6–42.1)	21.8 (20.7–22.9)	54.8 (53.5–56.1)	35.2 (34.0–36.5)	10.0 (9.1–10.8)
Nevada (4,426)	23.2 (21.6–24.9)	40.5 (38.3–42.7)	36.3 (34.1–38.4)	36.9 (34.9–39.0)	39.2 (37.0–41.3)	23.9 (21.9–25.8)	48.7 (46.5–50.8)	32.7 (30.5–34.8)	18.7 (16.8–20.5)
New Hampshire (7,020)	35.1 (33.6–36.6)	44.1 (42.4–45.8)	20.8 (19.2–22.4)	49.9 (48.2–51.6)	36.2 (34.6–37.9)	13.9 (12.5–15.3)	60.0 (58.2–61.7)	30.8 (29.1–32.4)	9.2 (8.0–10.5)
New Jersey (4,693)	32.6 (30.8–34.5)	44.6 (42.5–46.8)	22.7 (20.9–24.6)	47.9 (45.8–50.0)	38.9 (36.8–41.0)	13.2 (11.6–14.8)	55.3 (53.2–57.5)	31.5 (29.5–33.6)	13.1 (11.6–14.7)
New York (5,230)	30.6 (28.9–32.3)	47.1 (45.1–49.0)	22.3 (20.5–24.0)	46.2 (44.2–48.1)	41.6 (39.6–43.5)	12.3 (10.9–13.7)	54.6 (52.6–56.6)	33.0 (31.1–34.9)	12.4 (10.9–13.9)
Oklahoma (3,822)	23.6 (22.0–25.3)	41.9 (39.8–44.0)	34.5 (32.4–36.6)	32.8 (30.9–34.8)	39.5 (37.3–41.6)	27.7 (25.7–29.7)	57.2 (55.0–59.3)	32.8 (30.7–34.9)	10.0 (8.6–11.5)
South Dakota (7,488)	28.1 (24.5–29.7)	45.0 (43.2–46.8)	27.0 (25.3–28.6)	38.5 (36.8–40.3)	39.8 (38.0–41.6)	21.7 (20.1–23.2)	59.2 (57.5–61.0)	33.4 (31.7–35.1)	7.3 (6.4–8.3)
Tennessee (6,145)	26.3 (24.8–27.8)	34.5 (32.8–36.1)	39.2 (37.5–41.0)	35.5 (33.8–37.1)	34.3 (32.7–36.0)	30.2 (28.5–31.9)	54.8 (53.0–56.5)	28.7 (27.1–30.4)	16.5 (15.1–17.9)

**Abbreviation:** CI = confidence interval.

* Weighted percentages might not add to 100% because of rounding.

† Nondiet soda.

**TABLE 2:** Prevalence* of consumption of regular soda (i.e., nondiet) ≥1 times/day among adults, by age group, sex, race/ethnicity, and state — Behavioral Risk Factor Surveillance System, 18 states, 2012

	Regular soda consumption ≥1 times/day								
	
	Age group (yrs)†			Sex†			Race/Ethnicity†		
			
	18–34	35–54	≥55	Men	Women	White, non-Hispanic	Black, non-Hispanic	Hispanic	Other, non-Hispanic
									
State (no. respondents)	% (95% CI)	% (95% CI)	% (95% CI)	% (95% CI)	% (95% CI)	% (95% CI)	% (95% CI)	% (95% CI)	% (95% CI)
**Overall**§ (**11,3391)**	**24.5** (**23.0–25.9)**	**17.6** (**16.6–18.6)**	**10.2** (**9.6–10.9)**	**21.0** (**20.0–21.9)**	**13.5** (**12.8–14.2)**	**15.7** (**12.1–16.2)**	**20.9** (**19.1–22.7)**	**22.6** (**20.4–24.8)**	**10.7** (**8.4–13.0)**
**Range**	**18.3–47.4**	**12.0–33.0**	**6.8–20.1**	**15.3–36.8**	**9.0–28.5**	**8.9–30.0**	**10.8–37.1**	**12.2–43.5**	**4.9–34.2**
California (3,998)	18.5 (15.0–21.9)	13.8 (11.2–16.4)	6.8 (5.1–8.6)	17.2 (14.6–19.8)	9.3 (7.5–11.1)	8.9 (7.0–10.7)	10.8 (4.6–17.1)	21.7 (18.3–25.1)	7.9 (3.7–12.1)
Delaware (5,025)	26.9 (22.9–30.9)	17.4 (14.8–20.0)	10.7 (8.9–12.6)	21.3 (18.6–24.1)	14.4 (12.6–16.2)	16.3 (14.6–18.1)	21.2 (16.9–25.5)	25.0 (15.4–34.6)	—¶
Georgia (5,410)	30.6 (26.5–34.7)	23.8 (21.2–26.5)	14.9 (13.1–16.8)	26.0 (23.2–28.7)	20.1 (18.1–22.1)	21.4 (19.4–23.4)	22.5 (19.3–25.7)	35.8 (27.2–44.4)	18.4 (10.8–26.1)
Hawaii (7,152)	19.0 (16.0–21.9)	12.0 (10.0–14.0)	6.8 (5.6–8.1)	15.3 (13.3–17.2)	9.0 (7.6–10.3)	11.8 (9.4–14.1)	—	15.9 (10.3–21.4)	9.4 (7.9–11.0)
Iowa (3,277)	32.5 (27.6–37.5)	24.8 (21.6–28.0)	11.6 (9.9–13.4)	27.9 (24.7–31.0)	16.9 (14.6–19.3)	21.5 (19.5–23.5)	—	31.5 (19.3–43.7)	—
Kansas (5,616)	35.3 (31.5–39.2)	24.5 (21.7–27.4)	12.9 (11.3–14.5)	28.6 (25.9–31.2)	19.2 (17.3–21.2)	22.9 (21.2–24.6)	23.9 (16.1–31.6)	29.2 (21.2–37.3)	29.2 (17.8–40.7)
Maryland (5,760)	18.4 (13.7–23.1)	14.3 (11.5–17.0)	8.5 (7.0–10.0)	16.3 (13.4–19.3)	10.9 (8.9–12.9)	12.5 (10.4–14.5)	15.8 (12.0–19.5)	—	—
Minnesota (11,224)	28.3 (25.7–30.8)	19.1 (17.4–20.7)	8.5 (7.4–9.5)	23.1 (21.4–24.7)	13.1 (11.9–14.3)	17.2 (16.1–18.2)	19.8 (13.6–26.0)	26.8 (20.1–33.4)	19.5 (13.8–25.1)
Mississippi (7,242)	47.4 (43.5–51.4)	32.0 (29.3–34.7)	19.4 (17.7–21.1)	36.8 (34.1–39.5)	28.5 (26.5–30.6)	28.9 (26.9–31.0)	—	43.5 (28.2–58.7)	32.5 (18.3–46.6)
Montana (8,154)	24.1 (21.3–26.9)	18.5 (16.5–20.5)	7.9 (6.8–8.9)	20.3 (18.5–22.1)	11.5 (10.2–12.8)	14.4 (13.3–15.5)	—	23.7 (12.2–35.1)	23.7 (12.2–35.1)
Nebraska (11,709)	32.8 (30.3–35.3)	23.5 (21.6–25.4)	10.6 (9.5–11.7)	28.9 (27.1–30.6)	15.0 (13.8–16.3)	20.3 (19.2–21.4)	25.0 (18.5–31.5)	31.8 (26.5–37.1)	28.3 (20.5–36.2)
Nevada (4,426)	31.3 (27.0–35.6)	24.8 (21.6–28.1)	16.8 (14.0–19.5)	29.2 (26.1–32.3)	18.8 (16.4–21.1)	21.1 (18.8–23.3)	30.2 (21.7–38.7)	32.2 (27.5–36.9)	15.5 (8.9–22.0)
New Hampshire** (7,020)	25.4 (21.0–29.7)	13.0 (11.0–15.0)	7.0 (6.0–8.1)	17.5 (15.2–19.7)	10.6 (8.8–12.3)	13.7 (12.3–15.2)	—	—	17.4 (9.2–15.5)
New Jersey (4,693)	21.3 (16.9–25.8)	12.4 (10.1–14.7)	8.1 (6.6–9.6)	16.1 (13.6–18.6)	10.6 (8.5–12.6)	10.1 (8.4–11.8)	21.7 (16.1–27.4)	23.3 (17.8–28.7)	—
New York (5,230)	18.3 (14.7–22.0)	11.9 (9.8–14.0)	8.4 (6.7–10.1)	16.0 (13.7–18.3)	9.0 (7.4–10.6)	10.0 (8.6–11.4)	16.6 (11.6–21.6)	19.2 (14.8–23.6)	—
Oklahoma (3,822)	39.2 (34.4–43.9)	27.7 (24.5–30.9)	17.5 (15.4–19.5)	30.0 (26.9–33.1)	25.5 (23.0–28.1)	25.6 (23.4–27.9)	31.4 (22.9–40.0)	32.2 (24.2–40.2)	34.2 (26.7–41.6)
South Dakota (7,488)	33.3 (30.1–36.5)	22.9 (20.1–25.7)	11.4 (9.5–13.3)	29.1 (26.6–31.5)	14.4 (12.7–16.2)	20.2 (18.6–21.7)	—	31.8 (19.1–44.6)	32.6 (26.4–38.7)
Tennessee (6,145)	40.0 (35.9–44.1)	33.0 (30.1–35.9)	20.1 (18.2–22.0)	33.7 (30.9–36.5)	27.1 (25.1–29.1)	30.0 (28.2–31.8)	32.5 (27.6–37.4)	—	26.6 (14.3–38.8)

**Abbreviation:** CI = confidence interval.

* Weighted percentages might not add to 100% because of rounding.

† All values were p<0.05 by chi-square test.

§ Missing data: 0.5% for age and 2.8% for race/ethnicity.

¶ Data with sample sizes <50 or relative standard errors ≥30% not reported.

** Differences in regular soda consumption by race/ethnicity were not significant.

**TABLE 3:** Prevalence* of consumption of fruit drinks ≥1 times/day among adults, by age group, sex, race/ethnicity, and state — Behavioral Risk Factor Surveillance System, 18 states, 2012

	Fruit drink consumption ≥1 times/day								
	
	Age group (yrs)†			Sex†			Race/Ethnicity†		
			
	18–34	35–54	≥55	Men	Women	White, non-Hispanic	Black, non-Hispanic	Hispanic	Other, non-Hispanic
									
State (no. respondents)	% (95% CI)	% (95% CI)	% (95% CI)	% (95% CI)	% (95% CI)	% (95% CI)	% (95% CI)	% (95% CI)	% (95% CI)
**Overall**§ (**11,3391)**	**16.6** (**15.2–18.1)**	**11.0** (**10.1–11.8)**	**7.8** (**7.2–8.4)**	**12.3** (**11.4–13.1)**	**10.9** (**10.2–11.7)**	**8.1** (**7.6–8.6)**	**21.9** (**19.8–23.9)**	**18.5** (**16.4–20.6)**	**8.1** (**6.1–10.0)**
**Range**	**11.1– 28.7**	**6.1–18.8**	**6.0–10.8**	**8.3–20.0**	**5.6–17.4**	**5.9–13.8**	**9.2–30.5**	**8.4–33.8**	**3.8–25.5**
California¶ (3,998)	14.9 (11.3–18.4)	8.7 (6.7–10.7)	6.5 (5.0–7.9)	10.5 (8.3–12.7)	9.6 (7.7–11.4)	5.9 (4.4–7.5)	15.8 (6.9–24.6)	16.9 (13.7–20.1)	5.2 (2.3–8.1)
Delaware (5,025)	18.8 (15.2–22.3)	9.8 (7.7–11.8)	7.4 (6.1–8.7)	13.1 (10.9–15.4)	10.1 (8.5–11.7)	8.8 (7.5–10.2)	19.2 (15.2–23.2)	16.9 (8.8–24.9)	—**
Georgia¶ (5,410)	19.2 (15.8–22.6)	13.7 (11.5–15.9)	10.1 (8.7–11.5)	14.3 (12.2–16.5)	14.1 (12.3–15.6)	10.3 (8.9–11.6)	22.2 (19.0–25.5)	14.4 (8.1–20.7)	—
Hawaii (7,152)	14.5 (11.9–17.2)	8.9 (6.9–10.9)	7.5 (6.1–8.9)	11.4 (9.6–13.2)	8.7 (7.4–10.1)	7.2 (5.6–8.9)	—	18.3 (11.7–24.8)	9.2 (7.5–10.8)
Iowa¶ (3,277)	12.1 (8.6–15.6)	6.1 (4.3–7.8)	6.0 (4.7–7.2)	8.3 (6.4–10.3)	7.3 (5.5–9.0)	7.0 (5.7–8.2)	—	18.6 (8.0–29.1)	—
Kansas (5,616)	15.4 (12.3–18.4)	7.3 (5.6–9.1)	6.8 (5.6–7.9)	11.9 (9.8–14.0)	7.4 (6.2–8.6)	7.2 (6.2–8.2)	28.6 (20.0–37.2)	18.3 (11.6–24.9)	—
Maryland¶ (5,760)	15.0 (10.6–19.4)	9.8 (7.7–12.0)	7.0 (5.4–8.5)	11.9 (9.3–14.5)	8.8 (6.9–10.8)	8.2 (6.4–10.0)	13.4 (10.2–16.7)	17.0 (7.9–26.1)	—
Minnesota (11,224)	11.1 (9.3–13.0)	6.9 (5.8–8.0)	6.5 (5.6–7.4)	9.3 (8.2–10.4)	6.7 (5.7–7.7)	6.6 (5.9–7.2)	23.5 (16.5–30.5)	14.5 (9.6–19.4)	11.3 (7.1–15.5)
Mississippi¶ (7,242)	28.7 (25.1–32.4)	16.1 (13.8–18.4)	7.7 (6.7–8.7)	18.7 (16.4–21.1)	15.5 (13.8–17.2)	10.9 (9.3–12.4)	27.6 (24.7–30.5)	—	—
Montana (8,154)	12.6 (10.3–14.9)	7.7 (6.3–9.0)	6.7 (5.7–7.7)	10.5 (9.0–11.9)	6.9 (5.9–7.9)	7.5 (6.6–8.3)	—	—	25.5 (19.7–31.3)
Nebraska (11,709)	16.0 (14.0–18.1)	8.5 (7.1–9.8)	6.0 (5.2–6.8)	12.4 (11.0–13.8)	7.7 (6.7–8.7)	7.4 (6.6–8.1)	21.8 (15.3–28.4)	27.8 (22.5–33.1)	21.2 (14.0–28.4)
Nevada¶ (4,426)	26.6 (22.3–30.8)	18.8 (15.7–21.9)	12.1 (9.8–14.4)	20.0 (17.2–22.8)	17.4 (14.9–19.8)	11.5 (9.7–13.3)	28.7 (20.2–37.2)	33.8 (28.9–38.5)	15.5 (9.3–21.7)
New Hampshire¶ (7,020)	15.7 (11.9–19.5)	7.3 (5.8–8.9)	6.8 (5.8–7.9)	10.8 (9.0–12.6)	7.8 (6.2–9.4)	8.9 (7.7–10.0)	—	—	—
New Jersey (4,693)	19.8 (15.4–24.3)	12.3 (10.1–14.4)	9.2 (7.7–10.8)	13.9 (11.6–16.3)	12.4 (10.3–14.4)	8.7 (7.1–10.3)	25.9 (20.0–31.8)	24.4 (19.1–29.7)	—
New York¶ (5,230)	15.3 (11.8–18.8)	13.7 (11.1–16.3)	8.9 (7.0–10.8)	12.3 (10.0–14.5)	12.5 (10.4–14.5)	7.8 (6.6–9.1)	23.2 (17.3–29.2)	19.0 (14.4–23.6)	—
Oklahoma¶ (3,822)	16.4 (12.7–20.0)	8.9 (6.8–11.0)	5.4 (4.2–6.6)	10.5 (8.4–12.6)	9.6 (7.7–11.5)	7.0 (5.6–8.3)	23.5 (14.6–32.3)	26.1 (18.7–33.6)	10.6 (6.0–15.2)
South Dakota (7,488)	12.0 (9.8–14.2)	6.8 (4.9–8.6)	4.2 (3.1–5.3)	9.1 (7.5–10.8)	5.6 (4.5–6.7)	6.0 (5.0–6.9)	—	—	18.7 (13.3–24.2)
Tennessee¶ (6,145)	24.4 (20.8–28.1)	16.3 (14.0–18.6)	10.8 (9.3–12.3)	17.9 (15.6–20.2)	15.3 (13.5–17.0)	13.8 (12.4–15.2)	30.5 (25.7–35.3)	—	—

**Abbreviation:** CI = confidence interval.

* Weighted percentages might not add to 100% because of rounding.

† All values were p<0.05 by chi-square test.

§ Missing data: 0.5% for age and 2.7% for race/ethnicity.

¶ Differences in fruit drink consumption by sex were not significant.

** Data with sample sizes <50 or relative standard errors ≥30% not reported.

## Footnotes

* Additional information available at http://www.health.gov/dietaryguidelines/dga2010/dietaryguidelines2010.pdf.

† Additional information available at http://www.healthypeople.gov/2020/topicsobjectives2020/overview.aspx?topicid=29.

§ Additional information available at http://www.iom.edu/~/media/files/report%20files/2012/apop/apop_insert.pdf.

¶ Additional information available at http://www.cdc.gov/brfss/annual_data/annual_2012.html.

** Additional information available at http://www.cdc.gov/brfss/annual_data/2012/pdf/summarydataqualityreport2012_20130712.pdf.

†† Additional information available at http://www.cdc.gov/nchs/nhanes.htm.

§§ Additional information available at http://www.aacorn.org/uploads/files/AACORNSSBBrief2011.pdf.
